# Video playback versus live stimuli to assess quantity discrimination in angelfish (*Pterophyllum scalare*)

**DOI:** 10.3758/s13428-021-01738-8

**Published:** 2021-12-16

**Authors:** Luis M. Gómez-Laplaza, Robert Gerlai

**Affiliations:** 1 Department of Psychology, University of Oviedo, Plaza de Feijoo s/n, 33003 Oviedo, Spain; 2 University of Toronto Mississauga, Ontario, Canada

**Keywords:** Video playback, social stimuli, shoaling, quantity discrimination, angelfish

## Abstract

Video playback is a widely used technique for presentation of visual stimuli in animal behavior research. In the analysis of behavioral responses to social cues, presentation of video recordings of live conspecifics represents a consistently reproducible stimulus. However, video-recordings do not interact with the experimental subject, and thus this stimulus may be inferior in the social context. Here, we evaluated how angelfish (*Pterophyllum scalare*) respond to a video playback of conspecifics versus a live shoal of conspecifics. Using binary choice tests, subjects were presented different stimuli. Time spent close to one versus the other stimulus was considered an index of preference. We found angelfish to prefer a live shoal of conspecifics to an empty tank, and also the video playback of a shoal of conspecifics to a blank screen, although the level of preference in the latter was lower than in the former. These results indicate that video-playback of live conspecifics may be appropriate in angelfish, thus allowing manipulation of specific cues that angelfish may use in quantity discrimination. However, when we directly contrasted a live and a video recorded shoal, both having the same number of members, experimental fish preferred the live shoal. When the choice consisted of a live shoal of four conspecifics versus a video playback of a shoal of nine conspecifics no clear preference emerged. These results imply that video-playback has disadvantages in quantity discrimination studies with angelfish. Exploring procedural and/or technological parameters will verify the suitability of video-recording-based stimulus presentation for future use in angelfish.

Living in groups is widespread in fish species, presumably because it can confer various fitness benefits that outweigh the costs it may incur (Krause & Ruxton, 2002). The benefits include more efficient foraging (Day et al., 2001; Hintz & Lonzarich, 2018) and swimming (Marras et al., 2015; Miller & Gerlai, 2011), as well as better protection from predators (e.g., see Ioannou, 2017). The disadvantages include enhanced conspicuousness to predators (Botham et al., 2005), elevated parasitism (Poulin, 1999) and resource competition within the group (Maszczyk et al., 2014). These costs and benefits may vary with group size. Therefore, natural selection may have led to the evolution of the ability in fish to discriminate among differently sized groups. This assumption has been empirically confirmed in a variety of fish species by studies showing cognitive abilities of fish to discriminate between quantities, including number of shoal members (reviewed in Agrillo & Bisazza, 2018; Agrillo et al., 2017). But how do fish gauge group size?

A common way to investigate numerical abilities in fish has been to employ spontaneous dichotomous choice tests. In these tests, two aquaria, each containing a different number of conspecifics, are placed at the opposite sides of a central aquarium into which a test fish is placed. The time spent by the test fish in each zone close to the opposite sides of the test aquarium, i.e., in the proximity of the stimulus groups (shoals), is measured and generally considered as an index of preference of the test fish for one or the other shoal. The choice is regarded “spontaneous” as no training is involved, and thus the test relies upon inherent behavioral tendencies of the studied fish species. Such spontaneous choice tasks have been employed with a cichlid, the angelfish (*Pterophyllum scalare*), and the results have demonstrated excellent discrimination abilities (Gómez-Laplaza, 2006; Gómez-Laplaza, 2009; Gómez-Laplaza & Fuente, 2007), including the capability to discriminate quantities based on visual cues as well as the ability to discriminate based upon short-term memory of where the shoals were (Gómez-Laplaza et al., 2017; Gómez-Laplaza & Gerlai, 2011a, 2011b; Gómez-Laplaza & Gerlai, 2015; Gómez-Laplaza & Gerlai, 2016a, 2016b). Briefly, when conspecific shoals differing in numerical size (number of shoal members) were contrasted, angelfish chose the numerically larger one. Although this response has been generally robust, it has also been shown to depend upon a variety of factors, including non-numerical continuous variables (Gómez-Laplaza & Gerlai, 2012; Gómez-Laplaza & Gerlai, 2013a, 2013b).

In all these prior studies, live stimulus shoals were contrasted. However, live animals may behave inconsistently thus producing variable stimuli for the test subjects within and across tests. This aspect may create experimental error variation, and thus may reduce statistical power and reproducibility of results (Chouinard-Thuly et al., 2017; Gerlai, 2017; Gerlai, 2019). Furthermore, precise parametric control of most features of the live stimulus fish is impossible (e.g., see Gebuis & Reynvoet, 2012; Pekár & Kinder, 2020), and thus systematic analysis of the potential effects of such factors is difficult. A powerful, yet simple, method with which these issues may be addressed is the presentation of video-recordings.

Video playback has been widely employed in behavioral studies and has been found to provide excellent stimulus control and consistency, which, among some limitations, has been extensively discussed in the literature (see special issues dedicated to this topic in Acta Ethologica: Oliveira et al., 2000; and Current Zoology: Witte et al., 2017). Another advantage of video-recorded stimuli is that they reduce the number of live individuals needed in an experiment, an important consideration in the ethical use of animals in experimental research.

On the other hand, live stimulus fish may interact with the test fish in an ethologically relevant, “natural”, way, and thus may represent better stimuli compared to video-recorded fish. Perhaps for this reason, a positive response to the presentation of video images has not always been found, and diverse results have been reported that depended upon test contexts and species studied. For example, although in several studies similar preference and ways of responding to video stimuli and to live stimulus animals have been reported (e.g., Balshine-Earn & Lotem, 1998; Kodric-Brown & Nicoletto, 1997; Ord et al., 2002; Qin et al., 2014; Trainor & Basolo, 2000), other studies have failed to replicate findings between experiments using live versus video-recorded animals (e.g., D’Eath & Stamp Dawkins, 1996; Gonçalves et al., 2000; Patterson-Kane et al., 1997; Roberts et al., 2017).

The conflicting results, and the fact that both methods (presentation of live versus video-recorded animals) have pros and cons, make the question of which method is best suited for angelfish studies an empirical issue. The present study was designed to start testing the effectiveness of video playback, for the first time, as a tool for measuring quantity discrimination in angelfish, our model organism. Specifically, we examined the responses of angelfish to video-recordings of a shoal of angelfish versus to an actual (live) shoal of angelfish. A comparable response to these two different types of visual stimuli would validate the use of video-recordings in quantity discrimination studies with angelfish. It would also imply that video-recorded stimulus fish may be appropriate for future systematic dissection of visual cues to which angelfish respond in such experiments.

Briefly, in a series of dichotomous choice tests here we investigate how angelfish choose between presentation of: a) a live shoal of nine individuals versus an empty aquarium, b) two live shoals with the same number of individuals (nine) in each, c) a playback of a video-recorded shoal (with nine individuals) versus a blank screen, d) two playbacks of the same video-recorded shoal (with nine individuals), e) a live shoal versus a playback of a video-recorded shoal, both with the same number of individuals (nine), and f) a live shoal versus a playback of a video-recorded shoal, with the live shoal having a small number of individuals (four) and the video-recorded shoal having larger number of individuals (nine). In each of the above conditions, the contrasted stimuli (or lack of them) were presented simultaneously at the two opposite sides of the test tank, and the test fish were always naïve to the conditions, thus allowing the quantification of spontaneous preference. Conditions from a) to d) represent controls primarily to check for possible existence of side bias and whether test fish respond similarly to visual stimuli of fish (including live and video playback presented fish) versus no stimulus. These control conditions represented the first step for the validation of the use of video playback in this species. Conditions e) and f) were designed to evaluate the relative strength of preference for live versus video-playback presented stimulus fish.

## Material and methods

### Subjects and holding conditions

As in our previous studies (e.g., Gómez-Laplaza & Gerlai, 2020a, 2020b), juveniles of the freshwater cichlid species, angelfish (*Pterophyllum scalare*), measuring about 3.3 cm standard length, were obtained from a local supplier. Only juveniles of this sexually monomorphic species were studied to avoid potential confounding effects arising from courtship interactions. This diurnal cichlid fish from the Amazon river-basin is a highly social species that lives in groups (shoals), at least before reaching sexual maturity, likely as protection against numerous predators (Praetorius, 1932). Angelfish are usually found in well-illuminated mid/shallow water in their natural habitat, and are adapted to a dense aquatic vegetation, where vision is an important modality of perception. The anatomical (e.g., large plate-like eye size, the optic lobes being the largest component of its brain mass, White Jr., 1975) and behavioral features of the angelfish (e.g., the previously mentioned visual discrimination abilities), also suggest that this species possesses good visual acuity. A detailed account for the social behavior, communication and cognition of angelfish has been published recently (Brandão et al., 2021), implying that angelfish will be an excellent model organism for experimental behavioral studies.

Our experimental fish were initially maintained in glass aquaria (60 x 30 x 40 cm, length x width x depth) in groups of 18–20 individuals. Test fish and stimulus fish (which were used to elicit test fish behavior) were randomly chosen and were housed separately, without communication between different aquaria being possible. The holding aquaria had gravel substratum and were filled with dechlorinated water, kept at 26 ± 1°C using thermostat-controlled heaters. The water was continuously cleaned by external filters. Each aquarium was illuminated by a 15-W white fluorescent tube, and a 12:12-h light:dark cycle was maintained, with lights turned on at 0830 hours. The aquaria were lined on the outside with white cardboard, except for the front to allow observation by the experimenter. The fish were fed commercial flake food delivered to the water surface twice daily: at 1000 h and 1800 h. All fish were allowed a minimum of 2-week acclimation period before behavioral testing began.

### Test apparatus

**Fig. 1:**
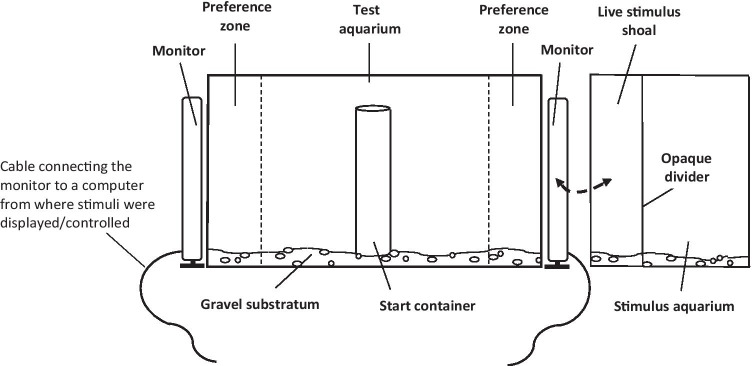
Experimental set up for dichotomous test, consisting of two monitors and a ~ 70-l tank. Videos were displayed on the two side monitors. In some conditions, the test aquarium was flanked by two small aquaria (only one is shown on the right of the figure) with live stimuli, or at one side by an aquarium with live stimulus and at the other side by a monitor. *Dashed line with arrows* indicates that monitors (where the video playbacks were displayed) and stimulus aquaria with live shoals were exchanged in some of the experimental conditions (see text). Monitors were connected to a computer

The experimental apparatus used to assess spontaneous preferences in binary choice tasks consisted of a test aquarium identical in all respects to the holding aquaria including their maintenance conditions. A stimulus aquarium, into which the live stimuli were placed, was positioned at both sides (short side) of the test aquarium (Fig. 1). The stimulus aquaria were also kept under the same conditions as the test aquarium, but were of smaller dimensions (30 x 30 x 40 cm depth). The side facing the test aquarium was of the same size as the short lateral sides of the latter (30 x 40 cm). A white opaque divider isolated a 10-cm compartment in the stimulus aquaria where the live stimulus shoals were presented. As with the holding aquaria, except for the front of the test aquarium, all exterior walls of the aquaria that were not adjacent to other aquarium walls were lined with white cardboard to prevent the fish from being influenced by external visual stimuli.

For presentation of video-recordings, the stimulus aquarium was replaced with a flat screen computer monitor (AOC model G2260VQ, 21.5”, refresh rate 75 Hz, full HD and 1920 x 1080 pixels resolution, China). The monitor was positioned flanking the testing aquarium (Fig. 1) and covered the entire width of the side glass. The monitor was connected to a computer (HP, Intel processor I5 7600, 240 Gb SSD Disc, NVidia Gigabyte GTX1050 Ti) from where the video playback was controlled. The computer also had an AOC monitor, identical to the stimulus presentation one.

Five vertical lines, drawn on the front and back walls of the test aquarium at a distance of 10 cm, divided the tank into six equal zones to facilitate measurements of the test fish’s movements and position. The two 10-cm zones closest to the stimulus aquaria/flat screens were considered to be the preference zones. Swimming activity of test fish was measured as the frequency (number of times) the fish crossed the lines drawn on the walls of the aquarium during the tests.

### Video recording

A shoal of nine angelfish, consisting of individuals randomly chosen, were placed in a stimulus aquarium under identical conditions, including illumination, space and background, as those during testing in live trials. After a 15-min acclimation period, this angelfish shoal swimming in the aquarium was video-recorded for 10 min with the experimenter absent to avoid interference with the stimulus fish. The recording was supported by the audio-visual media services of the University of Oviedo using a Canon EOS C100 MKII HD video camera (1920 x 1080 pixels resolution). The video was taken from the same viewpoint and distance from the shoal, about 30 cm away, as the test fish would view the live stimulus fish (while staying in the start container) before the tests commenced. The focal length of the camera was adjusted to the lateral side dimensions of the stimulus aquaria, so that the stimulus fish appeared life sized on the monitor. This digital video recording was transferred to the computer, and it was later replayed for the stimulus presentation. The computer was expanded with an additional graphic card and connected to all three monitors (two on the lateral sides of the test aquarium and one near the computer) where the video was displayed simultaneously. To adjust the size of the lateral sides of the aquarium to the area of the video visible on the monitor screens, the VLC software media player was sized accordingly. The computer, along with the corresponding monitor from where the presentation was viewed and controlled, was concealed behind a blind. A video length of 10 min was selected as angelfish have been found to express behavioral differences in choice tests within this time period (Gómez-Laplaza & Gerlai, 2020a, 2020b). After the video was recorded, the fish that served as the stimulus were returned to their home tanks in the laboratory. A sample of the video recording used as stimulus is located at http://hdl.handle.net/10651/59024

### Experimental procedure

In each trial, a single test angelfish, randomly chosen, was given a choice between two stimuli presented simultaneously. Stimuli were positioned in the stimulus aquaria, or presented on the screens of the computer monitors, on opposite sides of the test aquarium. Trials took place about 20 min after feeding in the morning. Before the start of a trial, the chosen number of fish that served as live stimulus shoals was randomly taken from the holding aquaria and was gently placed into the stimulus aquaria. The allocation of the stimuli to one end or the other of the testing aquarium was random for the first experimental subject, after which the location of the stimuli was counterbalanced across the experimental fish. Stimulus shoals were allowed a 15-min acclimation period, after which a randomly chosen experimental fish was individually placed in the center of the test aquarium via a transparent, open-ended, plastic cylindrical start container (7 cm in diameter; Fig. 1). Fish remained in the start container for 5 min, during which no stimuli were presented. After this period, the white opaque partitions separating the test aquarium from the stimulus aquaria were removed and the start container was then raised, releasing the fish to swim freely, and the trial commenced. The behavior of the experimental fish was recorded for a 10-min period using a video camera (Sony digital HD video camera recorder, model HDR-XR160E, China) concealed behind a blind placed 1.5 m away in front of the testing aquarium. The recordings were later replayed for analysis. Preference (choice) was defined as the time spent by the experimental fish in the 10-cm preference zones, i.e., within 10 cm from the wall adjacent to the stimuli on either side. Also, the first preference zone the experimental fish entered, the latency to enter the preference zones, the number of entries to these zones (frequency of entries), as well as overall swimming activity (as mentioned above) were quantified.

Identical procedures were followed when the stimuli consisted of a video playback of a shoal displayed on the screen monitors. In this condition, the stimulus aquaria were substituted by computer monitors also placed at opposite ends of the experimental tank. The position of the two video-recorded shoals was also alternated between the two sides of the experimental tank. Likewise, after a 5-min period in the start container, the white opaque partitions were removed and the two videos of the stimulus shoals played for 10 min, during which the experimental fish was released to swim freely.

Only one trial per day was conducted. After each trial, the aquaria were emptied and cleaned before being replenished with dechlorinated tap water. Stimulus shoals were randomly rearranged after each trial, so that each trial used a different stimulus fish set. None of the fish that served as stimuli were used as experimental fish and vice versa, and the different experimental conditions were randomly interspersed. Fourteen naïve angelfish were tested in each of the six experimental conditions, i.e., a total of 84 experimental fish were measured. At the end of the study, all experimental and stimulus fish were returned to the supplier.

### Experimental condition 1: a live shoal vs. an empty tank

This test was performed to assess whether angelfish prefer a shoal of nine conspecifics (placed in a stimulus aquarium) to an empty stimulus aquarium (placed in the opposite end of the test aquarium). Willingness to join a shoal of conspecifics when angelfish are introduced to a novel aquarium is a prerequisite to validate the type of choice paradigm we are employing in this study. As angelfish are a social species, we expected the experimental fish to choose the group of conspecifics over the empty tank. A sample of the recorded response of one of the experimental fish (9L vs. 0) is located at http://hdl.handle.net/10651/59024

## Experimental condition 2: two live shoals with the same number of members in each

The goal of this test was to examine whether fish exhibit a side bias. Experimental angelfish were individually presented with a binary choice consisting of two stimulus shoals of conspecifics, of nine members each, placed in the opposite sides of the test aquarium. We expected experimental fish not to exhibit a preference for either side.

### Experimental condition 3: a playback of a video-recorded shoal vs. a blank screen

With this test, we attempted to check whether angelfish can perceive and respond to the video recording, and whether they exhibit preference towards it. It represents a necessary condition for the use of video playback in this species. Given that angelfish has excellent vision and use visual stimuli in a number of behavioral contexts, we expected experimental fish to prefer the zone close to the monitor showing the video-recording of the nine conspecifics over the blank monitor screen placed on the opposite side of the test aquarium. A sample of the recorded response of one of the experimental fish (9V vs. B) is located at http://hdl.handle.net/10651/59024

### Experimental condition 4: two identical playbacks of a video-recorded shoal

In this condition, we exposed angelfish to a pair of video playbacks showing the same shoal of nine conspecifics to check for possible side bias when using video playbacks. As before, we expected to find no side bias, i.e., we expected equal preference for the two video playbacks. We note that in this experiment, we mirrored the video-recordings so that the orientation and movement of the video-recorded fish were always facing the same direction, i.e., the movement of the fish on the two sides of the test aquarium was synchronized.

### Experimental condition 5: a live shoal and a playback of a video-recorded shoal, both with the same number of individuals

This condition was designed to assess the relative effectiveness of the playback of video-recorded shoal versus the live shoal in eliciting an appetitive behavioral response (preference). For both the video-recorded and the live shoal, the shoal contained nine stimulus fish. If the video-recorded and live shoals are equally effective, angelfish should show a strong preference for both, and the level of preference should not show a significant difference between the two sides.

### Experimental condition 6: a live shoal and a playback of a video-recorded shoal, with the live shoal having a small number of individuals than the video-recorded shoal

This is another condition that should help clarify the effectiveness of video playback. It has been demonstrated that angelfish when given a choice between two shoals of conspecifics of different numerical size, they prefer the larger shoal and that the preference is usually ratio dependent (Gómez-Laplaza & Gerlai, 2011a; Gómez-Laplaza & Gerlai, 2016a). We assumed that angelfish prefer the live shoal to the video-recorded one. Here, we decided to create a conflict between this assumed natural preference for live conspecifics and the number of shoal members. That is, we presented the experimental fish with a choice between a live shoal consisting of four conspecifics and a video playback of nine conspecifics. If video playback is as effective as live conspecifics, according to our previous results, angelfish should prefer the video playback with the larger number of conspecifics over the smaller number of live fish.

### Statistical analysis

The time spent in the preference zones (sec) was considered as the main measure of the experimental fish’s preference for the corresponding stimulus. For each experimental fish, an index to quantify preference for one stimulus over the other was calculated as the proportion of time spent in the preference zone near the live shoal, relative to the total time spent in both preference zones. In each experimental condition, tests for normality (Shapiro–Wilk test) and for equality of variance (Levene’s test) were performed on the data before analysis. Data of latency to enter one and the other preference zone were not normally distributed, and they were *log* transformed to meet assumptions of parametric statistics. A one sample *t* test was used to investigate whether the observed preference index was significantly (*p* ≤ .05) different from chance (preference index = 0.5). The Holm–Bonferroni sequential correction method was used to correct for type I error resulting from multiple comparisons. Effect size for significant results was calculated by using Cohen’s *d*. A one-way ANOVA for independent samples was used to analyze the effect of the contrasts on preference, as well as on swimming behavior. Effect size for significant results was calculated using partial eta-squared (*ɳ*^*2*^*p*). In case of a significant effect, Tukey honestly significant difference (HSD) post hoc multiple comparison test was performed to determine where significant differences laid. Binomial probability tests comparing the number of fish initially choosing one stimulus or the other were used for each stimulus contrast. Frequency and latency scores were analyzed using paired *t* tests. All tests employed were two tailed. Statistical analysis was performed using SPSS Statistics version 23.0 written for the PC.

## Results

**Fig. 2:**
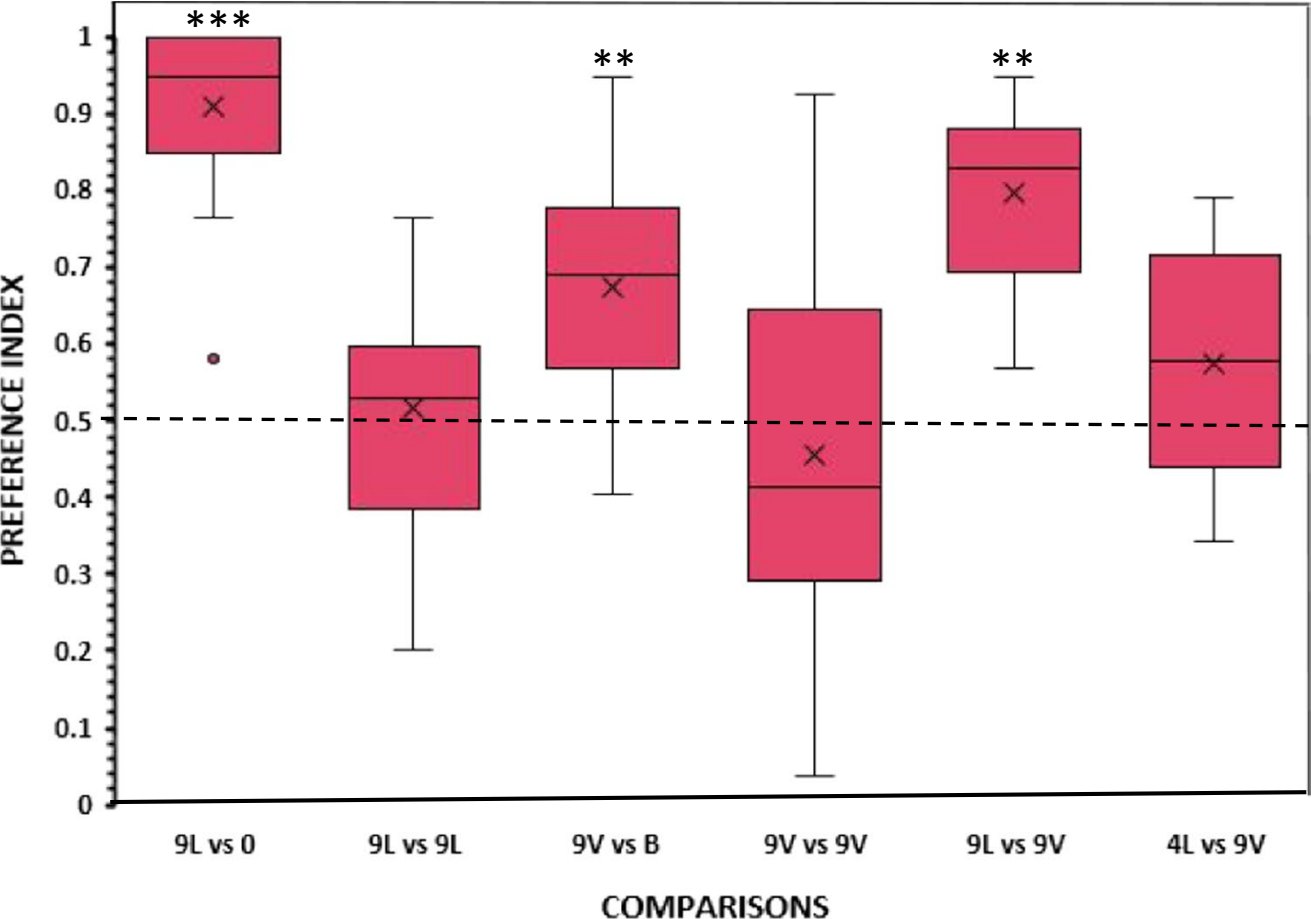
Proportion of time (preference index) spent by test fish in the preference zone close to the stimuli. *Box plots* show median (*horizontal line in the boxes*), 25% and 75% quartiles (*boxes*), and the lowest and highest values within the range of 1.5 times the respective quartiles (*whiskers*). *Open circle* shows outlier. *Blades* represent the mean proportion value. L = Live shoal, V = Video playbacked shoal, 0 = No fish (empty tank), B = Blank screen. Values above 0.5 indicate a preference for the numerically larger shoal or the live shoal. A significant departure from the null hypothesis of no preference is indicated by *asterisks*, ***p < .001, **p < .005

In the first experimental condition, when angelfish was presented with a nine-fish live shoal vs. an empty tank, test fish spent significantly more time than expected by chance in the preference zone close to the live fish (mean proportion of time (preference index) ± SEM: 0.9092 ± 0.0319; *t* test with Holm–Bonferroni correction *t*(13) = 12.839, *p* < .001, *d* = 3.433, Fig. 2). They preferred the zone close to the live-fish to the one near the empty tank, confirming that the choice task worked. The strong social preference is also highlighted by the fact that the first choice of 13 of the 14 experimental fish was the zone close to the live shoal (*p* = .002, see Table 1). The other behavioral parameters measured also confirm this preference, as the frequency of entries and the latency to enter the preference zone near the live stimulus fish were significantly higher and shorter, respectively, than for the zone near the empty tank (all *p* < .001, Table 1).

**Table 1:** Performance of angelfish (*N* = 14 in each comparison) when faced with the different stimuli and numerical contrasts. L = Live fish, V = Videotaped fish, 0 = No fish (empty tank), B = Blank screen

	First choice (out of 14 fish) ^a^			Frequency of entries^b^				Latency^c^				Swimming activity
	Larger/Live shoal	Smaller/No shoal/ Video	Binomial test	Larger/ Live shoal	Smaller/No shoal/Video	*t* test (*t*_13_)		Larger/Live shoal	Smaller/No shoal/Video	*t* test (t_13_)		
						value	Probability			value	Probability	
Controls												
9L vs. 0	13	1	P = .002*	4.57 ± 0.44	1.29 ± 0.32	8.891	P < .001*	7.86 ± 2.41	417.07 ± 54.04	8.259	P < .001*	22.14 ± 3.18
9L vs. 9L	6	8	*P* = .791	5.00 ± 0.71	5.36 ± 0.70	0.717	*P* = .486	90.71 ± 28.51	101.64 ± 39.83	0.013	*P* = .990	45.79 ± 6.86
9V vs. B	12	2	P = .013*	6.64 ± 0.88	4.00 ± 0.65	4.557	P = .001*	11.71 ± 4.65	109.50 ± 28.02	3.825	P = .002*	45.43 ± 6.47
9V vs. 9V	5	9	*P* = .791	5.07 ± 0.61	5.57 ± 0.55	0.563	*P* = .583	122.07 ± 37.50	42.71 ± 22.55	1.422	*P* = .179	45.50 ± 3.87
Contrasts												
9L vs. 9V	11	3	*P* = .057	5.50 ± 0.71	3.29 ± 0.40	3.012	P = .01*	23.93 ± 10.57	92.79 ± 15.31	2.714	P = .018*	50.93 ± 6.36
4L vs. 9V	8	6	*P* = .791	4.71 ± 0.42	5.14 ± 0.51	0.763	*P* = .459	82.29 ± 29.63	100.07 ± 30.25	0.447	*P* = .662	48.07 ± 4.77

Subjects were tested individually. Descriptive statistics includes mean ± SEM. The tests used to compare the scores are also included. ^a^Number of fish whose first choice was one or the other stimulus set. ^b^Frequency, number of times that subjects entered to the preference zones. ^c^Latency to enter the preference zone near one or the other stimulus set. * Statistically significant effects

In the subsequent condition with live stimulus fish on both sides (9 vs. 9 fish), experimental fish did not exhibit significant bias for either side of the aquarium where the stimuli were presented (mean proportion of time (preference index) ± SEM: 0.5172 ± 0.0405; *t*(13) = 0.424, *p* = .678, Fig. 2). That is, they did not spend more time in one or the other preference zones. In line with this finding, the other behavioral parameters measured (first choice, frequency of entries and latency to enter the preference zones) also confirmed the absence of preference for one over the other side having the live stimulus shoals of identical numerical size (all *p* > .05, Table 1). These results indicate that the apparatus and procedure are devoid of unknown confounds leading to side bias.

In experimental condition 3, when subjects were confronted with a choice between a playback of a video-recorded shoal of nine conspecifics versus a blank screen, angelfish showed a significant preference for the video playback (mean proportion of time (preference index) ± SEM: 0.6757 ± 0.0426; *t* test with Holm-Bonferroni correction, *t*(13) = 4.127, *p* = .004, *d* = 1.103, Fig. 2). The other behavioral parameters also revealed a significant preference for the video playback over the blank screen (all *p* < .05, Table 1), indicating that experimental fish clearly perceived the video-playback as an appetitive stimulus and responded to it.

Subsequently, another set of experimental angelfish were presented with two identical video-recordings showing the same shoal presented on the two sides of the testing aquarium. No significant difference was found between time spent on one versus the other side (mean proportion of time (preference index) ± SEM: 0.4555 ± 0.0632; *t*(13) = 0.704, *p* = .494, Fig. 2). Absence of significant differences was also revealed in the other behavioral parameters (all *p* > .05, Table 1). These results, thus, indicate that angelfish did not show any side bias when identical video playbacks were presented on the two sides of the test tank.

Perhaps the most critical condition in this study is in which a choice between a live shoal of nine angelfish and a video playback of a shoal of nine angelfish were presented. In this condition, experimental angelfish stayed longer in the preference zone close to the live fish. This preference was significantly greater than a random choice (mean proportion of time (preference index) ± SEM: 0.7973 ± 0.0292; *t* test with Holm–Bonferroni correction, *t*(13) = 10.169, *p* < .005, *d* = 2.718, Fig. 2). The first choice of experimental angelfish also showed a tendency for entering the zone near the live shoal rather than the zone near the videotaped shoal, as 11 of the 14 experimental fish made this choice (*p* = .057, Table 1). Both the frequency of entries and the latency to enter the zone close to the nine live fish (all *p* < .05, Table 1) confirmed that experimental angelfish preferred the shoal of nine live fish over the nine videotaped fish.

In the last experimental condition to assess the effectiveness of video playback to elicit shoaling behavior with the larger shoal, angelfish were exposed to a conflicting choice between a shoal of four live fish and a shoal of nine video-recorded fish. Although experimental fish exhibited a tendency to prefer the shoal of four live fish, this tendency was not significantly different from chance level performance (mean proportion of time (preference index) ± SEM: 0.5758 ± 0.0386; *t*(13) = 1.964, *p* = .071, Fig. 2). The other behavioral parameters also revealed a lack of significant preference for one over the other stimulus (all *p* > .05, Table 1).

Next, we examined how the performance of the experimental angelfish compared across all conditions. One-way ANOVA showed a significant difference in the preference indices across the six different conditions (*F*(5,78) = 16.709, *p* < .001, *ɳ*^*2*^_*p*_ = .517), and the Tukey HSD *post hoc* test revealed a significant difference in preference between the contrast of nine live fish vs. an empty tank as compared to all the other contrasts (all *p* ≤ .003) except the contrast of nine live fish vs. nine videotaped fish. In this latter contrast, preference for the nine live fish was not significantly different compared to what we found in the contrast of nine live fish vs. an empty tank (*p* = .431). Furthermore, in experimental condition 5 (nine live fish vs. nine videotaped fish), preference for live fish was significantly (*p* = .005) greater than in experimental condition 6 (four live fish vs. nine videotaped fish). Last, the magnitude of the response of angelfish to a videotaped shoal over a blank screen (condition 3) was found to be significantly (*p* = .006) greater than the response to two nine videotaped fish (condition 4).

A significant difference was also detected in swimming behavior among fish exposed to the six experimental conditions (*F*(5,78) = 3.682, *p* = .005, *ɳ*^*2*^_*p*_ = .191). This effect was mainly due to the lower swimming activity shown by experimental angelfish in the contrast between nine live fish versus the empty tank compared to swimming activity shown in any of the other contrasts (*p* ≤ .038; Tukey HSD test), with swimming activity in the latter contrasts not significantly differing among them (*p* > .05). The greater amount of time spent by the test fish near the nine live fish and the few approaches to the empty tank likely explain the lower activity exhibited by the fish in that condition (condition 1).

## Discussion

Video playback or computer animated stimulus delivery technologies have been an excellent tool in animal behavior studies (Chouinard-Thuly et al., 2017), particularly for species that use vision as their primary sensory modality (Gerlai, 2017). A large number of fish species are diurnal and indeed use vision as an important perceptual modality in a variety of behavioral contexts. Video or computer animation-based methods allow delivery of complex visual stimuli in a controlled and consistent manner, which may substantially improve replicability and reproducibility in fish research (Gerlai, 2019). In this study, we examined the effectiveness of playback of video-recordings of live shoals of angelfish with the goal of using the technology in future studies of quantity discrimination in this species. Since video-playback has never been used in angelfish, it was first necessary to verify whether the experimental set up was appropriate, i.e., whether it could elicit and detect visual stimulus choice/preference in angelfish. Results of experiments 1 and 3 demonstrated that the methodology worked properly in angelfish. Experimental angelfish responded to video playback and to live conspecifics and showed significant preference to these stimuli compared to a blank screen or an empty aquarium, respectively. The results confirm our previous findings that showed angelfish to prefer to stay close to conspecifics in a novel environment (Gómez-Laplaza et al., 2017; Gómez-Laplaza & Gerlai, 2011a). In our current study, we replicated this finding not just with live stimulus fish but also with using video playback of live conspecifics as a stimulus. Nevertheless, the strength of the response to the video-playback appeared to be lower compared to that elicited by the live conspecifics.

These are preliminary, but necessary, conditions to validate the use of video playback as a visual stimulus for the analysis of behavioral responses in animals (e.g., Chouinard-Thuly et al., 2017; Gierszewski et al., 2017; Roberts et al., 2019). Another precondition is the absence of spatial bias that could confound the results. Our results met this condition too. Experiments 2 and 4 found no side preference when the experimental fish were faced with a choice between two live shoals of equal number of conspecifics (experimental condition 2) or two identical video playbacks of conspecifics displayed on the monitors (experimental condition 4).

The significant preference for the video-recorded shoal versus the blank monitor is promising, but finding this preference less strong compared to the level of preference seen in the live shoal versus empty tank is noteworthy, as it suggests that perhaps live shoals represent a stronger stimulus. To directly address this question, we contrasted the video-recorded shoal with the live shoal in Experiment 5, and found the latter to be preferred to the former. The fact that angelfish showed equal choice between a live shoal of four conspecifics and a video-taped shoal of nine conspecifics (experiment 6) also supports the notion that angelfish prefer live to video-recorded shoals, because angelfish have been shown to exhibit strong preference for the shoal with larger number of members when the numerical ratio between the contrasted shoals is at least 2:1 (Gómez-Laplaza et al., 2017; Gómez-Laplaza & Gerlai, 2011a; Gómez-Laplaza & Gerlai, 2013b; Gómez-Laplaza & Gerlai, 2016a, 2016b). Other fish species too have been found to prefer live to video-recorded fish. For example, the freshwater darter, *Etheostoma barrenense*, showed a greater strength of preference for live stimuli than to video playbacks (Roberts et al., 2017). The zebrafish (*Danio rerio*) have also been shown to prefer to interact with live shoals of conspecifics over video-recorded shoals (Velkey et al., 2019). Last, the marine fish, the peacock blenny (*Salaria pavo*) failed to properly respond to video images of conspecifics (Gonçalves et al., 2000). In a study with guppies (*Poecilia reticulata*), using biomimetic robots it was also found that responses towards a live companion were stronger than towards the robot. This result was mainly attributed to the non-interactive behavior of the robot (Bierbach et al., 2018).

Our finding of angelfish to prefer live to video-recorded shoals, thus, is in line with the above results. Nevertheless, the reason for this preference is speculative at this point. Briefly, we do not know what aspects of the live shoal made this stimulus more preferred for angelfish than the video-recorded shoal. There may be several possible explanations. Although the size of shoal members, the density of the shoal, the number of shoal members within the shoal, the swim speed and other motor patterns of shoal members were all identical or very similar between the video-playback and live shoal, the live shoal was not shown through a monitor. Technical aspects of video-presentation, such as the RGB system providing color, the video-refresh rate of the playback, and even the light intensity may be idiosyncratic to the video-playback and may substantially differ from how light reflects back from a live shoal of fish and how this light is perceived by the experimental angelfish. In line with this reasoning, luminosity (D’Eath, 1998; Fleishman & Endler, 2000), depth perception (Zeil, 2000), motion (Rosenthal et al., 1996), and color perception (Fleishman et al., 1998) have all been identified as potential technical difficulties with video-presentations hindering resemblance to real animals (see also Schlupp, 2000). Notably, however, Carvalho et al. (2012) found that light intensity (ranging between 253 and 1446 lux) did not significantly affect aggressive behavior of angelfish. Another possible factor that may have negatively influenced the response of our experimental angelfish to the video-recorded stimulus is glare. Although computer monitors are designed to minimize glare, external light reflecting back from the monitor’s surface cannot be excluded as a confound. The last substantial difference between the video-playback and live shoal we re-emphasize here is that fish in the former cannot interact with the experimental fish. What among these, or perhaps other, differences between video-recorded and live stimulus fish may play roles in the behavioral responses of angelfish to these visual stimuli is an empirical question that will be experimentally addressed in the future.

Whether the video-recorded shoals, or even whether the live shoals are perceived by experimental angelfish as conspecifics is also an interesting question to which we do not have a clear answer yet. For example, live shoals may interact with the experimental angelfish but this fish have access only to visual stimuli. Olfactory, auditory, and perhaps most importantly, lateral line cues are all missing. The artificial nature of the presented stimulus is further complicated in case of the video-recorded shoal, as these fish cannot respond to or interact with the test fish. It is thus possible that angelfish does not perceive such stimuli as species specific cues but may simply explore these artificial stimuli. One aspect of such stimuli that may elicit exploration is movement alone. Moving objects have been found to elicit some level of preference in angelfish as well as other species (e.g., Alston & Humphreys, 2004; Krusche et al., 2010; Trick et al., 2003; see also Ware et al., 2015). Notably, in the current study, we found experimental angelfish to be more active in the presence of the video playback (versus a blank screen) than in the presence of the live shoal and an empty tank. We observed that the experimental angelfish, instead of staying with the videotaped shoal, repeatedly swam to explore the other side of the aquarium, implying a lack of strong shoaling response. A similar exploratory activity was observed in zebrafish, which instead of remaining close to conspecific images, i.e., shoaling, approached, explored and then swam away from moving objects presented on a computer screen (e.g., see Saif et al., 2013; Gerlai personal observation).

We note, however, that the superiority of live subjects to video-recordings is not inevitable. Females of the poeciliid fish *Poecilia formosa* exhibited a response to video images of a *Poecilia mexicana* male as strong as to live animals (Gonçalves et al., 2000), and males of pipefish (*Syngnathus typhle*) showed even stronger preference for the larger female shown in a video than for a smaller female shown live (Robinson-Wolrath, 2006). In other fish species, such as the tiger barb, *Puntius tetrazona*, the zebrafish, *Danio rerio*, the darter *Etheostoma zonale* and the sailfin molly, *Poecilia latipinna,* the videotaped stimuli were as effective as the real stimuli in eliciting a social response (Clark & Stephenson, 1999; Gierszewski et al., 2017; Qin et al., 2014; Roberts et al., 2017). It is thus possible that altering some technical parameters of the video-presentation of conspecific shoals may make this stimulus more preferred for angelfish too.

3D computer animation (Chouinard-Thuly et al., 2017; Gierszewski et al., 2017) and the use of virtual reality that mimics the sensory experience of real-world situations (Stowers et al., 2017) may also enhance the ecological and ethological validity of the artificially presented stimuli, and, by facilitating experimental control, may increase replicability and reproducibility of results (Gerlai, 2019). A similarly exciting methodological development in the area of stimulus control and delivery is the use of biomimetic robots that mimic the appearance and behavior of live animals (Bierbach et al., 2018). These robots can even be made interactive, that is, they can change their behavior in response to the actions of experimental animals (reviewed in Landgraf et al., 2021; and in Romano et al., 2019). Whether video-presentations, or other methods of stimulus deliver, could replace live stimulus fish presentation, however, remains an unanswered question, one which will require a set of systematic analyses. Such analyses perhaps could include computer animated images in which specific presentation features and characteristics of the images could be manipulated one by one (e.g., Gerlai, 2017).

Our current proof of concept study was not designed to explore such factors, but it did demonstrate a strong response to video-recorded conspecifics in angelfish. We regard this finding to be a promising start that may lead to the development of efficient and well controlled stimulus delivery methods for the analysis of quantity-estimating abilities as well as other cognitive functions of angelfish.
